# A Scoping Review on Accentuating the Pragmatism in the Implication of Mobile Health (mHealth) Technology for Tuberculosis Management in India

**DOI:** 10.3390/jpm12101599

**Published:** 2022-09-28

**Authors:** Jyotsna Needamangalam Balaji, Sreenidhi Prakash, Youngmok Park, Joon Sang Baek, Jaeyong Shin, Vasuki Rajaguru, Krishna Mohan Surapaneni

**Affiliations:** 1 Panimalar Medical College Hospital & Research Institute, Varadharajapuram, Poonamalle, Chennai 600-123, Tamil Nadu, India; 2 Division of Pulmonary and Critical Care Medicine, Department of Internal Medicine, Severance Hospital, Yonsei University College of Medicine, Seoul 03722, Korea; 3 Department of Human Environment and Design, Yonsei University, Seoul 03722, Korea; 4 Department of Preventive Medicine, College of Medicine, Yonsei University, Seoul 03722, Korea; 5 Institute of Health Services Research, Yonsei University, Seoul 03722, Korea; 6 Department of Healthcare Management, Graduate School of Public Health, Yonsei University, Seoul 03722, Korea; 7 SMAART Population Health Informatics Intervention Center, Foundation of Healthcare Technologies Society, Panimalar Medical College Hospital & Research Institute, Varadharajapuram, Poonamallee, Chennai 600-123, Tamil Nadu, India; 8 Departments of Biochemistry, Medical Education, Molecular Virology, Research, Clinical Skills & Simulation, Panimalar Medical College Hospital & Research Institute, Varadharajapuram, Poonamallee, Chennai 600-123, Tamil Nadu, India

**Keywords:** tuberculosis in India, mHealth, eHealth, mobile apps, TB management, chatbots, software applications, diagnosis, prevention, follow up, electronic applications, SMS based, mHealth interventions

## Abstract

*Background*: India continues to share a colossal count of the global tuberculosis load, with a perturbing 19% spring in the reported cases in 2021. With the National Tuberculosis Elimination Program (NTEP) consolidated to bring this epidemic to an end by 2025, the rapidly growing mobile health technologies can be utilized to offer promising results. Even though the implementation of this novel strategy is escalating around the globe, its triumph is still sub optimal in India. *Objectives*: This scoping review intends to explore the available mobile health (mHealth) technologies and analyse the effectiveness of the same for tuberculosis management in India. *Methods*: An elaborate search in electronic databases, such as PubMed and Google scholar, using the key terms and focussing from the year 2015, provided very broad results focussing on mHealth interventions and their utilisation in TB management in India. Further selection of the inclusive publications was carried out based upon the eligibility requirements as formulated for this review, pertaining to the objective of this study. *Results*: The collaborate search yielded a total of 858 scientific research papers. After the filtering of the obtained results, a total of 45 articles were selected to be analysed for this review. Published manuscripts, articles in peer review and abstracts from reliable databases were included to obtain vast range of information. *Conclusion*: The extensive literature search showed a preponderance of mHealth intervention studies focusing on TB treatment and drug monitoring. There exists a paucity of mHealth applications targeted to educate the public and intercept this infectious disease. The scientific articles reviewed and analysed in this scoping review strongly recommend the demployment of mHealth applications to achieve the target of eradicating TB by 2025 in India.

## 1. Introduction

Tuberculosis (TB) is an air borne transmissible infection caused by the Mycobacterium tuberculosis species which can affect other parts of the body too, but mainly exerts its effect on the lungs [1]. Preceded by COVID-19, TB is identified to hold the second position in the index of chief infectious killers, with an estimated population of 10 million people being infected in 2020 regardless of age and country. Among the nations with high TB burden, which contributes to two thirds of the global rise, India tops the list having the largest contribution [2]. As per the statistics of 2021, an average of 188 per 100,000 population are affected by tuberculosis of any form in India [3].

The control and prevention of the spread of diseases lies in devising beneficial schemes and proper implementation of public health strategies. The United Nations Sustainable Development Goals (SDG) aim towards good health and well-being [4] targets to reduce the global death rate of tuberculosis by 90% and bring down the total incidence of tuberculosis infection by more than 80% by no later than 2030 [5]. To curb the transmission and put an end to TB, the Government of India introduced the National Tuberculosis Programme in 1962, which was revitalized as the Revised National TB Control Programme and officially launched in 1997, covering Pan-India under this scheme by 2005, which aims to declare the cessation of TB infection by the year 2025 [6].

With the advent of mobile technologies and their ubiquitous usage, they surfaced as a dynamic equipment in healthcare [7]. Constructive use of mHealth has prospects for increased access to and practice of evidence-based medicine, educating healthcare consumers and vigorously engaging them in treatment and strengthening post-treatment care [8]. mHealth has the potential to furnish plausible solutions to wide ranging healthcare issues prevalent in India. The country is witnessing an unparalleled, rampant, and remarkable burgeoning of information and communication technology. This has enormously expanded the scope for mHealth interventions in India [9,10].

The utilisation of digital technologies (e.g., SMS based, smart phone applications, mobile voice calls) is exceedingly recommended by the WHO as an emerging opportunity to address the gaps in TB diagnosis, treatment, and care. mHealth interventions have the potential to find missing people with TB and promote TB care through case spotting, notification, diagnosis, reporting, treatment, drug monitoring and post-treatment care [11,12]. The intent of this scoping review is to deliver a recapitulation of the existing knowledge published in scientific literature and recent developments at the intersection of TB care and mHealth.

## 2. Methodology

The description of this study is written upon the exhaustive extraction of the information from scientific literature depicting the status of mHealth technology implications for tuberculosis in India from authentic sources, such as Google Scholar and PubMed from 28 July 2022 to 31July 2022. Each section of this scoping review was precisely drafted and cohered following the established guidelines of the PRISMA Extension for Scoping Review (PRISMA-ScR) [13].

### 2.1. Stage 1: Source of Information

A detailed search was performedin databases Google Scholar and PubMed to deduce publications concerned with the core objective of this research study. Scientific articles in the English language were selected from the year 2015.

### 2.2. Stage 2: Search Stratergy

**Figure 1:**
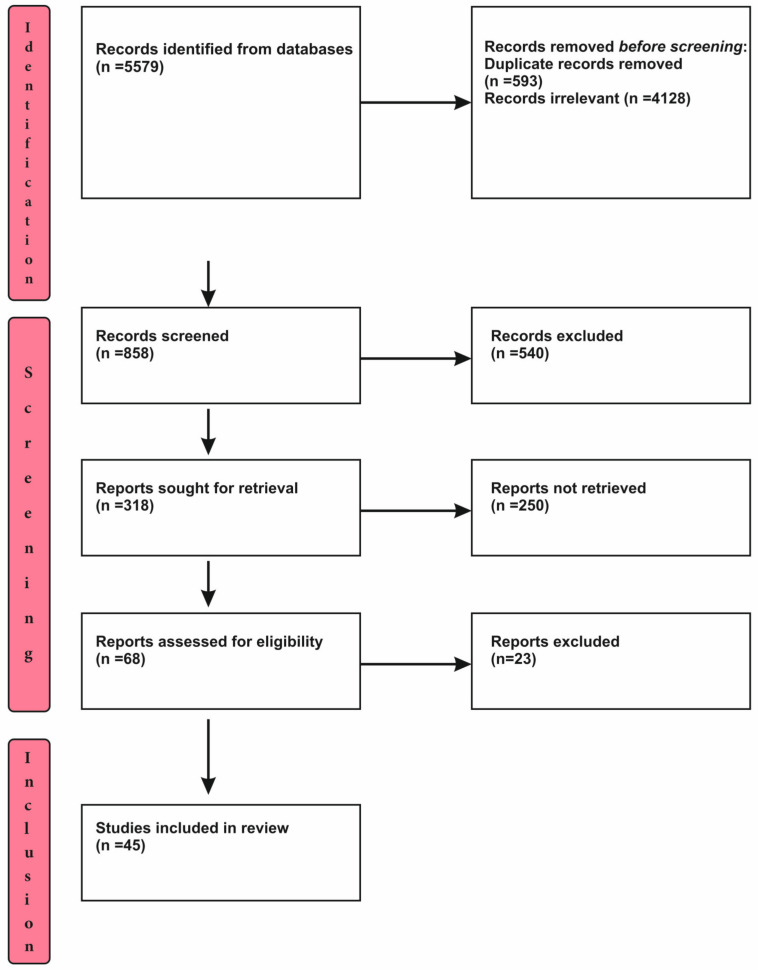
Process of Selection.

Relevant articles from well-grounded databases fromthe year 2018 were selected. Terms, such as ‘tuberculosis in India’, ‘mHealth’, ‘eHealth’, ‘mobile apps’, ‘TB management’, ‘chatbots’, ‘software applications’, ‘diagnosis’, ‘prevention’, ‘follow up’, ‘electronic applications’, ‘sms based’, and ‘mHealth interventions’, were used as key words to obtain the required results. The selection of literature from the search is presented in Figure 1.

### 2.3. Stage 3: Process of Selection

The process of slection includes three distinct steps, viz. Identification, screening and inclusion of studies. The process of selection has been detailed and depicted in the Figure 1.

### 2.4. Eligibility Criteria

Articles embraced for the content of this scoping view were selected based on the following inclusion/exclusion filtrations (Table 1).

### 2.5. Data Charting

An intricate data tabulation which includes alldependable variables was extracted and charted independently by the authors. Later it was meticulously reviewed, evaluated, and discussed by the team to conclude the finalised version of the data chart.

### 2.6. Data Items

After the collaborate appraisal of extracted data, segregation of relevant information and sorting under the columns of name of author, year, state/city, aim of study, type of study design, study population, type of intervention, name of intervention/app, languages available and result, was conducted.

## 3. Results

### 3.1. Selection of Source of Evidence

The aggregate count of 858 articles was selected for analysis. The publications were sorted following the eligibility basis. A total of 45 scientific literature works were chosen to be comprehensively studied for this review.

### 3.2. Characteristics and Results of Source of Evidence

The depicted table renders the conglomeration of relevant variables which were extracted and analysed for this review pertaining to the Indian setting (Table 2 and Table 3).

### 3.3. Summary of Charted Data

**Figure 2:**
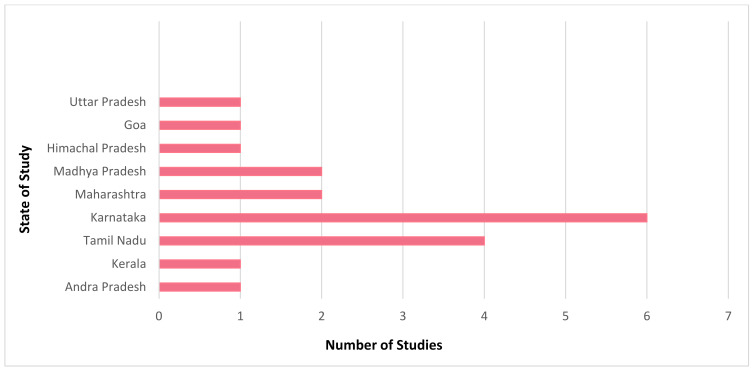
Selectednumber of studies conducted across different states in India.

**Figure 3:**
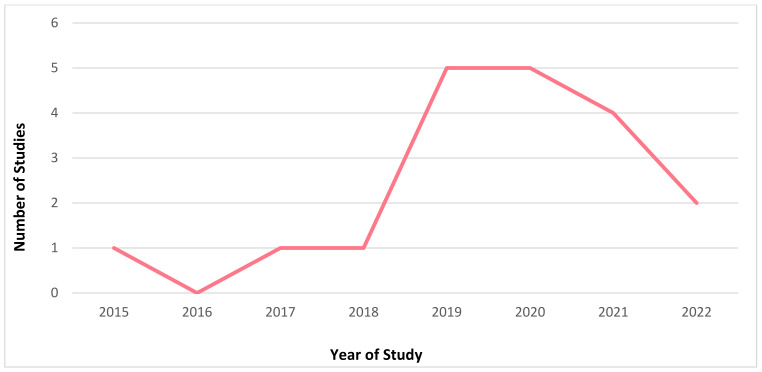
Frequency of published articles in a particular year selected for this review.

**Figure 4:**
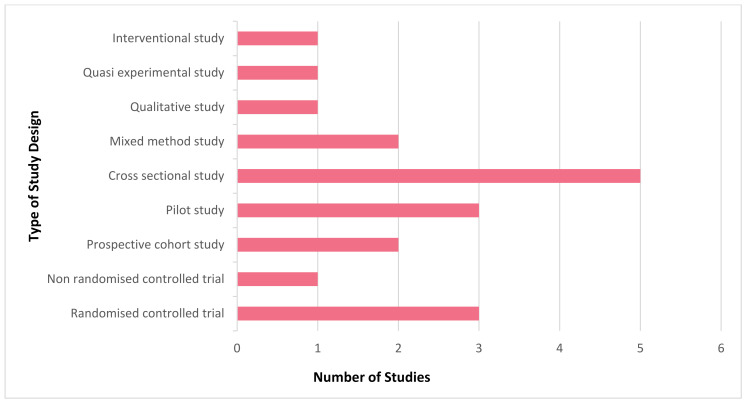
Different types of study designs of the articles included.

**Figure 5:**
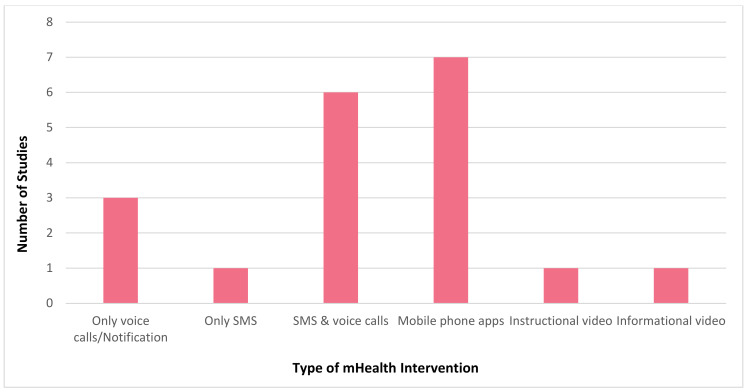
Type of mobile health (mHealth) interventions used in each study that was analysed for this review.

**Figure 6:**
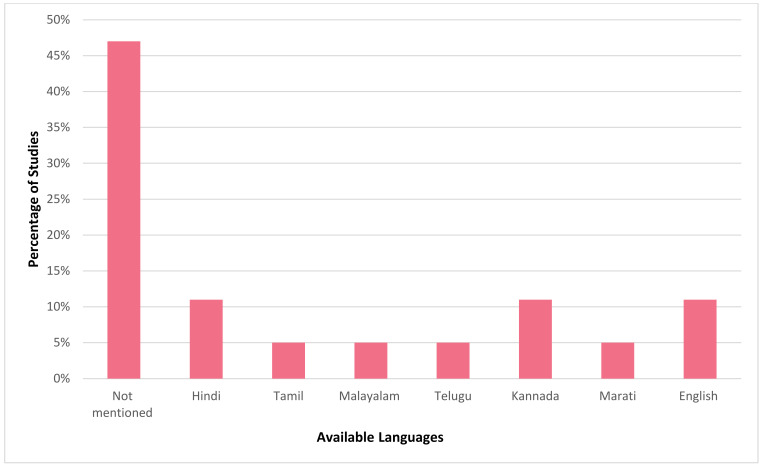
Predominant language in which the mHealth interventions chosen for this study that was available for use.

**Figure 7:**
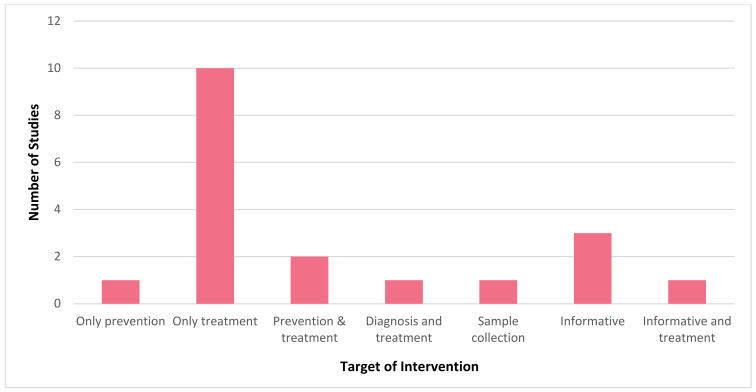
Target of moble health (mHealth) intervention used for tuberculosis management from the selected sources.

From the data as charted above, following elucidation can be made:

It is evident that mHealth interventions are widely used for tuberculosis management in India. However, the accessibility, utilisation and efficiency of this technological approach is yet to be substantiated clearly. Our scrutinised study of selected articles reveals that mHealth interventions for tuberculosis are implemented across India, with the highest number of studies being conducted in Karnataka (*n* = 6) followed by Tamil Nadu (*n* = 4) and Maharashtra and Madhya Pradesh (*n* = 2, each), with one study (*n* = 1) being conducted each in Andhra Pradesh, Kerala, Goa, Uttar Pradesh, and Himachal Pradesh. Figure 2 depicts the state where the studies included for this review were conducted in India. Figure 3 represents the number of publications from the year 2015 to 2022 that are selected for this review. From this, it is to be noted that most of these research studies wereconductedin the years 2019 and 2020 (*n* = 5, each). Our review encompassed a wide range of study design, including (i) randomised controlled trial (*n* = 3); (ii) non-randomised controlled trial (*n* = 1); (iii) prospective cohort study (*n* = 2); (iv) pilot study (*n* = 3); (v) cross sectional study (*n* = 5); (vi) mixed method study (*n* = 2); (vii) qualitative study (*n* = 1); (viii) quasi experimental study (*n* = 1); and (ix) interventional study (*n* = 1). Figure 4 portrays the different types of study design which were selected for analysis.In regard to the type of mHealth intervention that was used, it can be concluded that around 37% of mHealth interventions were mobile phone apps (*n* = 7), and approximately 32% comprised of conventional SMS and voice calls (*n* = 6). Certain studies have considered of only SMS (*n* = 1) or only voice calls/notifications (*n* = 3) as the mode of information transfer. Some studies have also used instructional (*n* = 1) and informational videos (*n* = 1) for promoting awareness. Figure 5 represents the type of mHealth intervention used for management of tuberculosis. Next, focussingon the language used in these mHealth interventions, Figure 6 shows the languages in which these interventions were demonstrated or used by the participants. The majority of the studies did not mention the language in which the intervention is available (*n* = 9), but a few applications have beendeveloped either in aregional language, such as Hindi (*n* = 2), Tamil (*n* = 1), Malayalam (*n* = 1), Telugu (*n* = 1), Kannada (*n* = 2), Marathi (*n* = 1) or English (*n* = 2), and some apps facilitate the use with the option to choose between English or their local language (*n* = 3). Finally, taking into consideration the target of these technical strategies, we categorized the interventions into those which concentrate only on tuberculosis (i) prevention (*n* = 1); (ii) treatment (*n* = 10); (iii) prevention and treatment (*n* = 2); (iv) diagnosis and treatment (*n* = 1); (v) sample collection (*n* = 1); (vi) informative videos (*n* = 3); and (vii) information and treatment (*n* = 1). From these, we can finalise that the majority of the mHealth interventions are being used to improve the adherence to treatment for tuberculosis. Figure 7 depicts the target of these mHealth interventions that are implemented for tuberculosis care.

The studies explored for this review primarily aimed at determining the usefulness of mobile health intervention in reminding TB patients to follow up and for facilitating the adherence to a TB regimen by the patient. A few studies also analysed the efficacy of telehealth intervention in promoting a healthy lifestyle for TB patients, such as cessation of smoking and alcohol consumption. Furthermore, the studies have extended themselves to ascertain the factors influencing the acceptance of DOTS in TB patients and healthcare providers and the outcome of adopting mHealth in anti-TB care (Table 2). The widely used types of mHealth intervention in the studies analysed are voice calls, mHealth applications in smartphones, SMS, followed by instructional videos (Table 3, Figure 4). The majority of the studies targeted the utility of mHealth intervention in enhancing the treatment and management of tuberculosis. SMS and voice call reminders have produced promising results in reinforcing patients’ adherence to an anti-tubercular drug regimen and have improved the treatment outcomes. Patient monitoring and follow up reminders have been made easier with the use of mHealth technology for patient care. Furthermore, the mHealth interventions received positive response from the patients and were viewed to be effective and affordable. Although the number of studies encompassed for this review that targeted the effectiveness of mHealth technology for prevention of tuberculosis is meagre, mHealth interventions have been claimed to promote healthy lifestyles among people, thus playing a significant preventive role. Informative video-based intervention aimed at disseminating knowledge about tuberculosis was found to have augmented the awareness of TB patients and the public in general about the importance of earlier diagnosis, available treatment options and course, together with its side effects, thereby accelerating the process of eradicating TB.

## 4. Discussion

There is massive hike in smartphone users across India, with 581 million users when collated to the last decade [15]. Utilization of this technology in low- and middle-income countries to provide superior quality healthcare services has been conceded as affordable and simple in recent years [33]. With the emergence of the COVID-19 pandemic, stern containment measures have remarkably reduced the movement and direct interaction of people, thus making the use of technology for health management inevitable [34]. mHealth technology has been implemented for the management of various health crises, such as HIV, cancer, tuberculosis, diabetes, hypertension, etc. [18,35]. With the National Tuberculosis Elimination Program functioning actively towards the termination of tuberculosis by 2025, mHealth serves as an advantageous approach to hasten the eradication process by imparting medical support, eventually fostering healthy lifestyles [36]. With a hefty burden of tuberculosis cases, mHealth is afeasible intervention to flatten the disease curve in India. Nonetheless, its acceptability and accessibility play a decisive role inlarge scale implementation.

Implementation of digital health technologies and adherence to the same for tuberculosis management has begun to escalate in India over the past few years, centralizing on patient care and targeted supervision [37,38,39]. From the reviewed articles, it is certain that numerous mHealth applications and mHealth schemes are available in India. However, lack of proper regulation and implementation has led to its suboptimal utilisation. The data analysed from the selected literature strongly endorsed the employment of mHealth strategies for effective TB management [14,15,21,26]. This will reduce the load on healthcare systems burdened with high disease prevalence, low workforce, restricted financial support, and higher numbers of rural residents [40]. It is also to be noted that there are increasing internet facilities in rural areas, favouring the Government of India in its initiative to magnify the adaptability and accessibility of digital health services among the general population [41]. Studies have presented encouraging results stating effective communication through mHealth applications enhances TB management, in contrast to ineffective patient–medical staff interaction [42].

Smartphone applications aid early diagnosis and encourage patients to attend regular follow ups, thus leading to better prognosis [16,25]. A randomised controlled trial conducted in Goa has proved the success of mobile tele-counselling interventions in fostering smoking cessation in TB patients [17]. Moreover, 99DOTS, a cell phone-based intervention, is subject to non-adherence because of poor cell phone accessibility and cellular signal [19]. A mobile phone app with a Quick Response (QR) tracking system is found to be more efficacious in patient monitoring [22]. Scientific data have also discovered that patients have an optimistic attitude towards implementation of mHealth strategies [24]. After meticulous analysis it was explored that among the all the interventions, patients preferred video-based Direct Observation Treatment (DOT) followed by SMS and voice calls [26,30,32]. Contradicting this finding, a cross sectional study conducted in Pune put forth that the result acceptability of text messages was found to be minimal among TB patients [27]. Less exposure to newer technologies contributed to midwives being hesitant towards mHealth technologies [29]. Although the factors contributing to adherence or non-adherence to mHealth interventions is not well established, crucial factors involved include availability, accessibility, and availability of mHealth technology, literacy, employment status, signal, and network coverage [25,26,27].

The main reason for substandard treatment outcome is non-adherence to therapy protocol [43]. mHealth related reminders in the form of interactive text messages or voice calls that are human assisted instead of whole automation are deemed to be the most welcoming intervention [44,45]. Investigations have unveiled that there exists a prolonged delay of 55 days in initiating TB treatment after confirmation of diagnosis [46]. Early closure of loopholes in TB care assists in decreasing its spread and incidence. This could be accomplished by early notifications via digital health technologies [47,48]. Video Observation Treatment is established to have an upper hand over Direct Observation Treatment in that it does not require any appointment with the provider and can be deployed outside business hours [49,50].

Healthcare workers and community workers, such as Accredited Social Health Activist (ASHA) groups, serve as a connecting link between patients and medical professionals. It is important to make sure that even these workers are efficiently trained in using mHealth technologies to help the patients. Hence, it is essential to address the issues of these workers, such as illiteracy, misdiagnosis, security and privacy issues, work automation and queries regarding reliability [51,52,53]. To broaden the application and usability of mHealth strategies, it is imperative to inculcate technological awareness through proper educational feeds and consistent guidance by well-trained staff [54]. Appropriate policy formulation, financial management and data governance structure to vanquish under reporting or over reporting of cases are proposed for upgrading awareness of people and to boost the resolution to use and accept digital health services [55,56,57].

## 5. Knowledge Gaps

The findings of our review suggest that the quantity and quality of data available pertaining to mHealth and TB care are insufficient to arrive at a definite conclusion. A majority of mHealth interventions have been tested in urban cities of India. There is not enough evidence advocating the applications of mHealth interventions in rural settings. Studies have focused more on the outcomes of the interventions. There is a lack of knowledge about factors that are instrumental in leading to the desired outcome. None of the reported studies have disclosed the software architecture on which the mHealth apps were built. This restricts the duplication and large-scale application of similar interventions. The documented studies are not in line with behavioural theoretical frameworks. Interventions subjected to behavioural theory have efficacious outcomes. Lastly, the application of chatbot technology in TB care remains an understudied topic.

## 6. Limitations

The scheme of this scoping review was to focus attention on the use of mHealth technology for tuberculosis care in the Indian setting. Thus, all other manuscripts originating from any other country were excluded. Published scientific works only from the year 2015 onwards were taken into consideration, as older articles may not be on level with the recent developments in the field of technology and healthcare. Moreover, manuscripts published only in the English language wereselected for descriptive analysis. Finally, publications of only two databases, Google Scholar and PubMed, were selected and reviewed for writing the content of this study.

## 7. Directions for Future Research

We strongly recommend that robust scientific studies evaluating theefficacy of mHealth interventions by virtue of aptly drafted and sampled studies are crucial for instituting the on-field fitness of mHealth initiatives. Research targeting rural populations is required as more than half the population of the country resides in rural India.

Moreover, it appears worth discovering the sustainable financing of mHealth technologies used for TB, scrutiny of TB diagnosis equipment stocks and TB drug forecasting. Furthermore, mHealth app developers and researchers should ensure that adequate information about the app is provided for its replication and large-scale implementation. Existing trends and advancements in technology, such as developments in the use of machine learning, artificial intelligence, and block chains, have the capacity to unfurl novel opportunities in mHealth. To transfigure these opportunities to real benefits, vigorous research and a multifaceted approach is needed to tackle issues of data governance mechanism, interoperability, and sustainability of digital health over the long term.

## 8. Conclusions

Our findings show that there are finite research articles on availability, accessibility, and utilisation of mHealth by healthcare workers and patients for prevention, diagnosis, treatment, and management of tuberculosis. Technological interventions, such as smartphone applications, mobile voice calls, SMS, and educative videos have been claimed to produce a profound, positive impact on disease outcome. Researchers have asserted the feasibility and acceptability of mHealth interventions for drug adherence in a resource limited setting. Moreover, the studies have voiced the need to tailor the existing mHealth interventions that best suit the Indian population. Our review suggests that the need of the hour is to generate authentic evidence on user acceptability, efficacy, feasibility, and affordability of mHealth interventions directed towards TB management strengthening. A rational approach would be to encompass an implementation research element into the current and propounded digital health initiatives to reinforce origination of proof for TB care and management strengthening on strategically significant sequels.

## Figures and Tables

**Table 1:** Criteria for inclusion and exclusion of articles.

Parameters	Inclusion	Exclusion
**Language**	English	All other languages
**Year**	From 2015	Before 2015
**Type of study**	Quantitative, qualitative and mixed methods	
**Country**	India	All other countries
**Publication status**	Articles under peer review, grey literature, indexed journals, abstracts	
**Study theme**	mHealth interventions for Tuberculosis management	Manual methods of management
**Intervention**	Mobile health apps, Short Message Service (SMS)	Physical modes of diagnosis, management, or treatment

**Table 2:** Characteristics of the selected articles.

S. No.	Author	Year	State/City	Aim of Study	Study Design	Study Population
1	Majella et al. [14]	2021	Puducherry	To evaluate the use of mobile voice calls to remind Tuberculosis (TB) patients regarding follow up	Randomised controlled trial	Newly diagnosed TB patients
2	Nagaraja et al. [15]	2020	Karnataka	To observe missed doses during treatment in TB patients with or without mobile application	Prospective cohort study	694 adult TB patients
3	Zhang et al. [16]	2020	Bhopal and Indore	To study the feasibility of the smart phone app	Pilot study	18,706 patients
4	Fernandes et al. [17]	2021	Goa	To determine the effectiveness of tele-counselling to quit smoking in TB patients	Randomised controlled trial	80 individuals
5	Velayutham et al. [18]	2015	Chennai	To ascertain the usefulness and affordability of mobile interface in TB notification	Pilot study	184 private medical practitioners
6	Thomas et al. [19]	2020	Chennai and Vellore	To determine the factors affecting the acceptance of 99DOTS (Directly Observed Treatment, Short course) in TB patients and healthcare providers	Qualitative study	93 patients
7	Tyagi et al. [20]	2020	Not specified	To explore the preferences of healthcare providers on the content of information and communication technology for TB	Mixed method study	24,949 private practitioners
8	Gupta et al. [21]	2020	Shimla	To analyse the outcomes of mHealth interventions in tuberculosis care	Randomised control trial	312 patients
9	Navin et al. [22]	2018	Chennai	To investigate the application and utility of an mHealth intervention for supporting tuberculosis treatment	Prospective cohort study	TB patients
10	Pande et al. [23]	2017	Manipal	To investigate the utilisation of a smartphone app among clinicians for tuberculosis	Cross sectional study	101 clinicians
11	Kumar et al. [24]	2019	Bangalore	To explore the usage of mobile apps and mHealth support regarding tuberculosis management among patients	Cross sectional study	185 TB patients
12	Santra et al. [25]	2021	Delhi	To assess the potency of mHealth intervention on medication follow up among TB patients	Quasi experimental study	220 TB patients
13	Rao et al. [26]	2022	Bengaluru and Ujjain	To analyse the usage of mobile phones for the anti-tuberculosis regimen among patients	Cross sectional study	351 TB patients
14	Cox et al. [27]	2019	Pune	To investigate the access to mobile phones and mHealth technology for management of tuberculosis	Cross sectional study	136 Indian TB patients
15	Jose et al. [28]	2022	Kerala	To analyse the acceptance of mHealth interventions for the anti-tuberculosis treatment.	Cross sectional study	100 TB patients
16	Kodali et al. [29]	2021	Andhra Pradesh	To study the approved practice of mHealth technology among auxiliary nurse midwives	Mixed method study	272 auxiliary nurse midwives
17	Holzman et al. [30]	2019	Pune	To investigate the feasibility and utilisation of mobile apps for video-based Directly Observed Therapy (DOT) among TB patients	Pilot study	25 TB patients
18	Shivalli et al. [31]	2019	Karnataka	To access the efficacy of mobile based video instructions on sputum expectoration in order to enhance the quality and quantity of sputum sampling for tuberculosis patients	Non-randomised controlled trial	7341 presumptive pulmonary tuberculosis PTB patients of which 311 patients were diagnosed with TB
19	Nagaraj et al. [32]	2019	Bengaluru	To understand the adherence of patients to mobile based educational videos for regimen care to enhance tuberculosis treatment	Interventional study	100 TB patients

**Table 3:** Information on mHealth interventions in the studies analysed.

S. No.	Type of Interventions	Name of app/Interventions	Available Languages	Target	Result
1	Voice calls [14]	Not mentioned	Tamil	Treatment	Mobile voice calls are effective interventions to reduce Pretreatment loss to follow-up PTLFU among TB patients
2	Mobile phone app [15]	Kill TB	Not mentioned	Treatment	mHealth app enhances the patient’s treatment adherence and improves treatment outcomes
3	Mobile phone app [16]	Not specified	Not mentioned	Treatment and diagnosis	The use of smart phone application can boost diagnosis and follow up of patients
4	Voice calls [17]	Not mentioned	Not mentioned	Prevention	Mobile tele-counselling is a feasible intervention to encourage smoking cessation
5	Voice based system for notification [18]	Mobile interface in TB notification (MITUN)	Not mentioned	Prevention and treatment	The effectiveness of Mobile interface in TB notification (MITUN) was found to be suboptimal
6	Short Message Service (SMS) and voice calls [19]	99DOTS (Directly Observed Treatment, Short course)	Not mentioned	Treatment	Poor cell phone accessibility, cellular signal and literacy are some of the factors contributing to non-adherence to 99DOTS
7	Mobile phone app [20]	Think TB	Not mentioned	Informative	Providers had inherent preferences for instructional content
8	Short Message Service (SMS) and voice calls [21]	Not specified	Hindi	Treatment	These mHealth technologies are easier to access and provide effective means of communication when physical presence couldnot be offered for every reminder.
9	Mobile phone app with unique Quck Response (QR) scanning [22]	TB track	Not mentioned	Treatment	A Quick Response (QR) tracking system provides unique monitoring of patients. Reminders for medication intake, reporting symptoms and tracking of progress are effectively well monitored.
10	Mobile phone app [23]	LearnTB	English	Informative	The overall utility of the app was very good which advocates the fact that mHealth technologies are more efficient and accessible.
11	Short Message Service (SMS) and voice calls [24]	Not specified	English	Treatment and prevention	Mobile communication received a high optimistic response among the participants. Reminders regarding medications, medical appointments, follow up and general preventive information was well appreciated to be received as SMS and voice calls
12	Short Message Service (SMS) and voice calls [25]	Not specified	Hindi	Treatment	mHealth technologies offer a supportive hand for daily regimen reminders of TB patients and weekly follow up. Future studies are essential in making these interventions accessible to all
13	Short Message Service (SMS) and voice calls, video based Directly Observed Therapy (DOT) [26]	Not specified	Not mentioned	Treatment	Short Message Service (SMS) and voice call preference was higher among frequent mobile users and video-based Directly Observed Therapy (DOT) was on high demand as it is cost effective, requires less travelling and convenient for users.
14	Short Message Service (SMS) reminders [27]	Not specified	Not mentioned	Treatment	Acceptability towards text messages was suboptimal among patients. Literacy, employment status and frequency of mobile usage were found to affect the acceptance rate
15	Short Message Service (SMS) and voice calls [28]	Not specified	Malayalam and English	Treatment	Voice calls were preferred to SMS reminders. However, a quarter of the participants accepted the usage of mobile phones for effective treatment reminders.
16	Mobile phone app [29]	Not specified	Telugu and English	Informative	mHealth acceptance was found to be less among the midwives, which can be attributed to less technological awareness and less exposure to newer methods of information transmission.
17	Mobile phone app [30]	Emocha Mobile Health Inc.	Marathi and English	Treatment	Video based Directly Observed Therapy (DOT) are identified to be feasible and affordable means of follow up of treatment and it can be used as a reliable monitoring tool among TB patients
18	Instructional videos [32]	Not specified	Kannada	Sample collection	SMS and voice calls are rapidly used for TB management in India. However, implementation of higher standard technologies may be costly and adhering people to these interventions may be a challenge as very fewstudies have been conducted in regard to video explanations.
19	Informational video [32]	Not specified	Kannada	Informative and treatment	The video-based technological support was found to have greater adherence and enhanced the awareness of TB patients about the importance of diagnosis, side effects and available treatment facilities to promote better health conditions.

## Data Availability

The data that support this study are available upon request from the corresponding author.
